# Altered peripheral lymphocyte subsets in untreated systemic lupus erythematosus patients with infections

**DOI:** 10.1590/1414-431X20198131

**Published:** 2019-04-15

**Authors:** Zhimin Lu, Jing Li, Juan Ji, Zhifeng Gu, Zhanyun Da

**Affiliations:** Department of Rheumatology, Affiliated Hospital of Nantong University, Nantong, People's Republic of China

**Keywords:** Systemic lupus erythematosus, Lymphocyte subsets, Infection, C-reactive protein, Procalcitonin

## Abstract

The leading cause of death in systemic lupus erythematosus (SLE) patients is infection. The objective of this study was to evaluate the distribution of lymphocyte subsets in untreated SLE patients with infections. This was a cross-sectional study. Data from January 2017 to May 2018 were collected. Flow cytometry was used to measure the peripheral lymphocyte subsets including CD3^+^T cells, CD4^+^T cells, CD8^+^T cells, CD19^+^B cells, CD3^-^CD16+CD56NK cells, and CD3^+^CD16+CD56NKT cells in 25 healthy controls and 52 treatment-naive SLE patients, among whom 13 were complicated with infections. Association between the lymphocyte subsets and infections was further analyzed. SLE patients with infections (n=13) showed a significantly higher incidence rate of fever (84.6 *vs* 28.2%) and serositis (84.6 *vs* 23.1%), increased level of erythrocyte sedimentation rate (60.5±30.1 *vs* 37.4±27.1 mm/h), serum C-reactive protein (CRP) (102.7±94.9 *vs* 9.4±14.9 mg/L), procalcitonin (PCT) (1.07±0.08 *vs* 0.16±0.13 μg/L), and lower blood hemoglobin (Hb) (93.0±20.5 *vs* 110.4±16.0 g/L) level compared with non-infection patients (n=39) (all P<0.05). In comparison with non-infectious SLE patients (387.9±261.6/μL), CD4^+^T cells count decreased significantly in infectious SLE patients (217.8±150.4/μL) (P<0.05), and it was negatively correlated with infection-related indicators including PCT (r=−0.573, P=0.041) and CRP (r=−0.596, P=0.032) levels. Our findings suggested that abnormalities of peripheral lymphocyte subsets were related to the immune disorder of lupus itself, regardless of immunosuppressive treatment. Monitoring lymphocyte subsets, especially CD4^+^T cells, may be helpful for identifying the presence of infection in SLE patients.

## Introduction

Systemic lupus erythematosus (SLE) is a typical autoimmune disease characterized by complex immunological abnormalities and highly diverse clinical manifestations. Patients with SLE are often in a major immunosuppressive state caused by the immune disorder and application of immunosuppressive agents. Infectious complications, especially of the skin, and respiratory and urinary systems, develop in up to 50% of SLE patients (1,2), and serious infection remains a main cause of hospitalization and mortality (2,3). Infectious pathogens have also been shown to play a role in the pathogenesis of and increased disease activity in SLE (4).

It has been demonstrated that immune disturbances of lupus itself and use of immunosuppressive drugs are both important risk factors for infections in SLE patients, but the underlying mechanism remains elusive (5,6). It is not clear whether the increased burden of infections seen in SLE patients relates to the immune disorder underlying the disease, treatment with immunosuppressive agents, or the interplay between these factors (6).

Since there is scant evidence of infection analysis of new-onset SLE patients without treatment, the main aim of the present study was to address whether inherent immune disturbances are an independent risk factor for the development of infections. As we know, peripheral lymphocytes play an important role in anti-infection immune response and abnormal status accounts for a bad ending in infection diseases (7). We mainly investigated the major lymphocyte subsets in untreated SLE patients and analyzed their relation to infections. As a secondary aim, we explored some credible makers for identifying the presence of infection in SLE patients.

## Material and Methods

### Patients and controls

Fifty-two SLE patients with no history of corticosteroids or immunosuppressive drug use were recruited from the Department of Rheumatology of the Affiliated Hospital of Nantong University from January 2017 to May 2018. These SLE patients had not taken glucocorticoids or immunosuppressive agents when they went to our hospital for diagnosis and treatment. The SLE patients fulfilled the 1997 SLE classification criteria revised by American College of Rheumatology (8). Twenty-five age- and sex-matched healthy controls (HC) were enrolled in this study. Patients complicated with tumor or other autoimmune diseases were excluded. This cross-sectional study was approved by the Ethics Committee of the Affiliated Hospital of Nantong University, and written informed consent was obtained from all subjects. Infection was considered definite or probable according to culture results. A definite bacterial infection was diagnosed if an organism was identified on culture or microscopy. In the absence of a pathogenic bacteria being identified, a probable bacterial infection diagnosis was made on the basis of a combination of clinical findings, review of imaging studies, laboratory finding such as white blood cell count, and a response to only antibiotic therapy.

The baseline demographic and clinical data were collected from hospital records and reviewed by experienced physicians. The data included age, gender, and duration. Routine laboratory investigation included white blood cell (WBC), blood hemoglobin (Hb), blood platelet (PLT), erythrocyte sedimentation rate (ESR), serum levels of C-reactive protein (CRP), procalcitonin (PCT), serum concentrations of complement factors C3 and C4, anti-dsDNA using an immunoblotting technique, serum IgG, IgM, and IgA, and 24-h urinary protein levels.

Disease activity was measured using the Systemic Lupus Erythematosus Disease Activity Index (SLEDAI) score according to the medical records (9).

### Sample collection and preparation

Peripheral blood of SLE patients and healthy controls was collected. The blood of SLE patients was collected prior to therapy with glucocorticoids and immunosuppressive agents. Four milliliters of heparinized blood was diluted with the same volume of phosphate-buffered saline (PBS). Peripheral blood mononuclear cells (PBMCs) were prepared by Ficoll-Plaque (Pharmacia, Sweden) density gradient centrifugation (400 *g*, 18°C, 30 min), washed in RPMI 1640 culture medium (Gibco, USA) twice, and then resuspended at a concentration of 2×10^6^ cells/mL.

A typical panel of markers used to identify the major subset of lymphocytes included CD3, CD4, CD8, CD19, and CD16+56. Freshly isolated and cultured PBMCs were suspended in PBS. For the staining of surface antigens, cells were incubated with FITC-conjugated anti-CD3, APC-conjugated anti-CD4, PE-conjugated anti-CD8, APC-conjugated anti-CD19, and PE-conjugated anti-CD16+56 (all from BD Bioscience, USA). Mouse anti-human FITC-, PE-, and APC-conjugated IgG1 were used as isotype controls. All cell samples were assayed by a FACSCalibur flow cytometer (BD Bioscience) and the acquired data were further analyzed using FCS Express V3 (De Novo Software, Canada) analysis software. Flow cytometric results are reported as positive percentages. Trucount tubes (BD Bioscience) were used to determine the absolute number. The percentages and counts of the lymphocyte subsets including CD3^+^T cells, CD4^+^T cells, CD8^+^T cells, CD19^+^B cells, CD3^-^CD16+CD56NK cells, and CD3^+^CD16+CD56NKT cells, as well as CD4/CD8 ratio were measured.

### Statistical analysis

All data were analyzed using SPSS version 17.0 software (IBM, USA). Means±SE or interquartile ranges are reported for numeric values with normal and non-normal distribution, respectively. Categorical variables are reported as frequency and percentage. Clinical characteristics of infection and non-infection SLE patients were compared using Student's *t*-test, Wilcoxon Rank-Sum test, or the chi-squared test, as appropriate. Pearson correlation analysis was performed to evaluate the correlation between variables of peripheral lymphocyte subsets and infection-related indicators in SLE patients with infection. P values <0.05 were considered statistically significant.

## Results

### Clinical characteristics

A total of 52 patients diagnosed with SLE were included; 13 patients were complicated with infections. Patient characteristics are reported in Table 1. Classification of infections were as follows: pneumonia (n=6), upper respiratory tract infection (n=5), gastrointestinal tract infection (n=1), and septicemia (n=1). The median age of SLE patients with infection was 31.0±9.6 years (female/male: 12/1) and that of non-infection SLE patients was 36.4±13.5 years (female/male: 37/3) (P>0.05). The mean age of HC was 32.3±8.9 years (female/male: 22/3). Disease activity was calculated using SLEDAI score. There was no significant difference of the mean SLEDAI score between the infection and non-infection group (12.3±3.4 *vs* 10.8±5.3, P>0.05). The average disease duration of the infection group was 6.9±13.0 months, which was significantly shorter than that in the non-infection group (19.8±29.4 months) (P<0.05). SLE patients with infections showed significantly a higher incidence rate of fever (84.6 *vs* 28.2%) and serositis (84.6 *vs* 23.1%) compared with non-infection patients (both P<0.05). There were no differences in the incidence rates of new rashes, arthritis, nephritis, or central nervous system, gastrointestinal or cardiac involvement between the two groups.

**Table 1:** Characteristics of untreated systemic lupus erythematosus (SLE) patients with and without infection.

Features	Non-infection (n=39)	Infection (n=13)	P
Age (years)	36.4±13.5	31.0±9.6	0.192
Gender (F / M)	37 / 3	12 / 1	1.000
Disease duration (months)	19.8±29.4	6.9±13.0	**0.035**
Clinical manifestations					
Fever	11 (28.2%)	11 (84.6%)	**<0.001**
New rashes	28 (71.8%)	6 (46.2%)	0.178
Arthritis	16 (41.0%)	3 (23.1%)	0.406
Serositis	9 (23.1%)	11 (84.6%)	**<0.001**
Nephritis	12 (30.8%)	5 (38.5%)	0.864
CNS involvement	2 (5.1%)	0 (0%)	1.000
Gastrointestinal involvement	4 (10.3%)	1 (7.7%)	1.000
Cardiac involvement	1 (2.6%)	1 (7.7%)	0.434
Laboratory data					
ANA antibody positive	39 (100%)	13 (100%)	1.000
Anti-SSA antibody positive	28 (71.8%)	9 (69.2%)	1.000
Anti-SSB antibody positive	10 (25.6%)	3 (23.1%)	1.000
Anti-SM antibody positive	16 (41.0%)	7 (53.8%)	0.420
Anti-dsDNA antibody	374.1±321.3	515.0±358.6	0.204
AnuA	98.2±85.6	119.4±95.1	0.489
WBC(×10^9^/L)	4.6±2.2	5.0±3.0	0.541
Hb (g/L)	110.4±16.0	93.0±20.5	**0.003**
PLT (×10^9^/L)	185.4±85.4	186.6±106.1	0.968
ESR (mm/h)	37.4±27.1	60.5±30.1	**0.013**
CRP (mg/L)	9.4±14.9	102.7±94.9	**0.004**
PCT (μg/L)	0.16±0.13	1.07±0.08	**0.001**
IgG (g/L)	17.9±9.4	20.9±6.5	0.335
IgA (g/L)	2.6±0.9	3.0±1.3	0.275
IgM (g/L)	1.3±0.7	1.5±1.2	0.679
C3 (g/L)	0.45±0.21	0.41±0.21	0.616
C4 (g/L)	0.087±0.057	0.092±0.071	0.812
24-h urinary protein (g)	1.0±2.1	1.4±1.2	0.565
SLEDAI	10.8±5.3	12.3±3.4	0.348

Data are reported as means±SD or number and percent within parentheses. CNS: central nervous system; ANA: antinuclear antibodies; dsDNA: double-stranded DNA; AnuA: anti-nucleosome antibodies; WBC: white blood cells; Hb: blood hemoglobin; PLT: blood platelets; ESR, erythrocyte sedimentation rat; CRP: serum levels of C-reactive protein; PCT: procalcitonin; Ig: immunoglobulin, C3: complement 3; C4: complement 4; SLEDAI: systemic lupus erythematosus disease activity index. Significant differences (P<0.05) are shown in bold. Student's *t*-test, Wilcoxon Rank-Sum test, or the chi-square test were used as appropriate.

Compared with non-infection patients, SLE patients with infection had a significantly higher serum level of ESR, CRP, and PCT (all P<0.05), the mean Hb level of infection patients was significantly lower (P<0.05). There were no significant differences in the levels of WBC, PLT, complement, antiantibodies, or urinary protein levels between the two groups.

### Distribution of lymphocyte subsets

The lymphocyte subset results of these three groups including HC, non-infection SLE patients, and infection SLE patients are summarized in Table 2. In comparison with HC, the absolute number of CD3^+^T cells, CD4^+^T cells, CD8^+^T cells, NK cells, and NKT cells were significantly down-regulated in SLE patients with and without infection (all P<0.05). The CD4/CD8 ratio was lower in the two SLE patient groups compared with HC (both P<0.05).

**Table 2:** Distribution of lymphocyte subsets in untreated systemic lupus erythematosus (SLE) patients with and without infection.

	Healthy controls (n=25)	Non-infection (n=39)	Infection (n=13)	P		
				Non-infection *vs* HC	Infection *vs* HC	Non-infection *vs* infection
Lymphocyte	2031.6±537.6	1114.2±625.5	745.2±372.3	0.000	0.000	0.051
CD3^+^T (%)	75.4±3.9	73.8±9.6	73.6±11.7	0.366	0.605	0.952
CD4^+^T (%)	42.9±4.7	34.3±9.9	28.9±8.8	0.000	0.000	0.085
CD8^+^T (%)	31.4±7.5	36.6±10.1	40.5±13.9	0.029	0.041	0.272
CD19^+^B (%)	10.8±4.4	18.5±9.4	19.4±10.9	0.000	0.017	0.783
NK (%)	13.4±4.9	6.0±3.7	5.0±4.4	0.000	0.000	0.421
NKT (%)	4.8±1.7	3.31±2.26	3.92±2.44	0.011	0.262	0.520
CD3^+^T (μL)	1536.3±443.1	830.6±510.9	531.9±262.6	0.000	0.000	**0.05**
CD4^+^T (μL)	870.1±248.1	387.9±261.6	217.8±150.4	0.000	0.000	**0.031**
CD8^+^T (μL)	645.1±249.5	415.7±327.6	281.1±151.7	0.004	0.000	0.161
CD19^+^B (μL)	218.3±102.3	203.8±146.9	162.5±137.4	0.667	0.165	0.378
NK (μL)	267.9±111.9	62.2±44.9	36.5±36.3	0.000	0.000	0.068
NKT (μL)	100.7±55.6	36.6±26.7	23.2±26.7	0.000	0.001	0.188
CD4/8	1.4±0.6	1.03±0.47	0.84±0.45	0.001	0.001	0.196

Data are reported as means±SD. Significant differences are shown in bold type (Student’s *t*-test). HC: healthy control.

**Figure 1:**
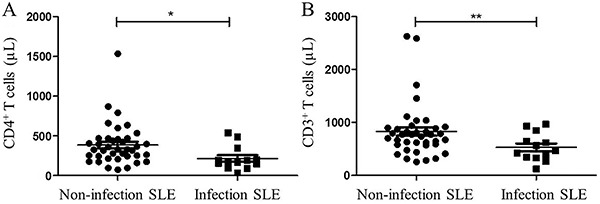
Distribution of (**A**) CD4+ and (**B**) CD3+ T cell numbers between the two groups of systemic lupus erythematosus (SLE) patients. *P<0.05, **P=0.05 (Student's *t*-test).

This study confirmed that CD4^+^T cells in peripheral blood decreased significantly in infectious SLE patients compared to non-infectious SLE patients (217.8±150.4 *vs* 387.9±261.6/μL, P<0.05, Figure 1A). The peripheral CD3^+^T cells were marginally down-regulated in SLE patients with infections (531.9±262.6 *vs* 830.6±510.9/μL, P=0.05, Figure1B).

In addition, we observed a similar tendency in absolute number of NK cells, but it failed to reach statistical significance.

### Correlation between peripheral CD4^+^T cells

Correlation analysis showed that in infectious SLE patients, the absolute number of peripheral CD4^+^T cells was negatively correlated with serum PCT (r=-0.573, P=0.041, Figure 2A) and CRP (r=-0.596, P=0.032, Figure 2B). No significant correlation was found between peripheral CD4^+^T cells with conventional inflammatory markers of ESR.

**Figure:**
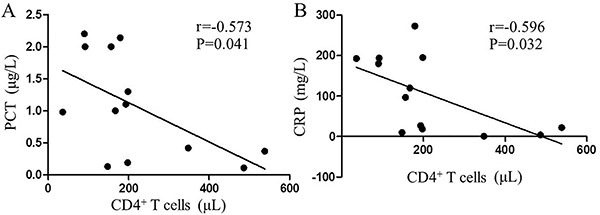
Associations of peripheral CD4+ T cells with (**A**) procalcitonin (PCT) and (**B**) C-reactive protein (CRP) in infectious systemic lupus erythematosus (SLE) patients. Data are reported as absolute number of peripheral CD4^+^ T cells (μL). Pearson correlation analysis was used for statistical analyses.

## Discussion

SLE is a heterogeneous disease characterized by highly diverse clinical manifestations and complications. Infection complications are known to be a major cause of hospitalization and mortality in SLE although the five-year survival rate in SLE has dramatically increased recently due to earlier diagnosis (1–3,10).

In terms of infection complications, Danza and Ruiz-Irastorza (11) reported that disease activity, high anti-DNA titers, low complement levels, nephritis, leucopenia, prednisone doses over 7.5–10 mg/day, and high doses of methylprednisolone and cyclophosphamide were well-recognized risk factors for infection. However, a study from a multiethnic lupus cohort showed that specific TNF variants and leukopenia influenced the risk of developing pneumonia, regardless of immunosuppressive therapy (12). Research from the Hospital Universitario de Santander suggested that anemia, lymphopenia, hypocomplementemia, and especially the activity of the disease are risk factors for invasive fungal infections in SLE patients (13). In addition, Balbi et al. (14) reported that SLE patients with nephritis and high cumulative doses of corticosteroids may be more prone to developing tuberculosis, especially in endemic countries.

Multivariate analysis identified that SLE flare was an independent predictor of infection-related mortality among SLE patients, while immunosuppressive medications and corticosteroids were not risk factors (2). Discrepancy in the results of these studies may be explained by differences in treatment regimen, as many SLE patients investigated in the majority of previous studies had already been treated with long-time and high doses of immunosuppressive agents, which may influence immune responses.

It is not certain whether the increased rate of infections itself in SLE patients relates to inherent immune disturbances or the relevant medicine given for disease control. To investigate the important role of disease activity itself and inherent abnormal immune status in infection susceptibility in SLE patients, this study analyzed the distribution of lymphocyte subsets in patients with no glucocorticoid or immunosuppressive agent therapy as well as its correlation with infections.

It is well known that the imbalance of activated lymphocyte subsets correlates with the development of SLE (15,16). In SLE patients, the most common changes in lymphocyte subsets are a reduction of CD4^+^ T cells and imbalance of CD4/CD8 ratio (16,17). Boomer et al. (7) observed extensive depletion of splenic CD4, CD8, and HLA-DR cells in patients who died from active severe sepsis, suggesting that internal immune immunosuppressive status accounts for a bad ending, just as materialist dialectics holds “external factors play a role only through internal factors”.

There are only a few reports on lymphocyte subsets comparisons between SLE patients with and without infection, especially in untreated patients. Wolfe and Peacock described that lymphopenia and low CD4^+^T cell count are risk factors of pneumocystis pneumonia in connective tissue diseases (18). Wu et al. (19) reported that the CD4^+^T cell number and the CD4/CD8 ratio as well as immunoglobulin G level were lower in SLE patients with infection than in those without infection. It is not clear whether decreased CD4^+^T cell number and CD4/CD8 ratio relate to the nature of the autoimmune disease or the immunosuppressive agents.

To exclude the influence by the immunosuppressive agents, we enrolled new-onset SLE patients untreated with corticosteroids or immunosuppressive drugs. In our study, we observed that the absolute number of CD3^+^T cells, CD4^+^T cells, CD8^+^T cells, NK cells, and NKT cells were remarkably down-regulated in SLE patients compared with HC, which is consistent with previous studies (16,17). As we know, antibody production by B cells requires help from CD4^+^T cells, so defects in CD4^+^T cells may lead to severe immunodeficiencies. As no immunosuppressive agent was used, we presumed that the altered CD4^+^T cells related to the nature of the autoimmune disease and the infections. Further studies should expand the group numbers to perform a further multivariate analysis to search for factors related to CD4+ T cell numbers.

On the other hand, current evidence supports the hypothesis that infections may play the role of environmental triggers of various autoimmune diseases in genetically prone individuals (4,20). Infectious pathogens in the 13 infectious SLE patients may play a role in the progress of these genetically predisposed individuals. We supposed the infectious SLE patients had significantly shorter disease duration partly due to the trigger of the infectious pathogens. The patients with infection showed a significantly higher incidence rate of fever. The severe clinical manifestations such as fever may drive patients to see doctors. As a result, the patients may be treated earlier and have shorter disease duration.

Due to an abnormal immunological response, the clinical manifestations of the infections can be atypical. Therefore, careful inspection and monitoring are warranted to avoid misdiagnosis. Some studies reported CRP and PCT levels were higher in infection than flare in SLE patients (21,22). A meta-analysis showed that PCT levels are significantly higher in Asian SLE patients with infection (23). Our data also showed that both CRP and PCT levels were higher in SLE patients with infection. We further analyzed the correlation between peripheral CD4^+^T cells and infection-related indicators in infectious SLE patients. As shown in Figure 2, peripheral CD4^+^T cell count was negatively correlated with serum PCT and CRP.

In our study, we verified that SLE patients with CD4^+^T cell depletion were more prone to develop infections. Considering the individual limitations of each biomarker, we propose the use of these indices together. Clinical testing for lymphocyte subsets is potentially useful for identifying the presence of infection in SLE patients, which may allow physicians to have a more accurate diagnosis.

Our study had several limitations. One of the main limitations was that this study was performed in a single center. Also, the number of patients was small because of the selection criterion of untreated SLE patients. In addition, lymphocyte subsets were evaluated only once after admission and no follow-up was conducted. Another limitation was the heterogeneity of infections and the criteria for diagnosing different types of infections.

In summary, our findings suggested that abnormalities of the CD4^+^T cell subset related to the nature of the autoimmune disease might make the patient more susceptible to infectious pathogens, regardless of treatment with immunosuppressive agents. In addition, CD4^+^T cell count negatively correlated with infection-related indicators. Monitoring lymphocyte subsets, especially CD4^+^T cells, may be helpful for identifying the presence of infection in SLE patients.
